# Obituary

**Published:** 1909-11-27

**Authors:** 


### Obituary.

The death is announced of Lieutenant-Colonel F. J. Crawford, M.D., I.M.S., at sea on November 5. He was a Fellow of the University of Madras, and had only recently been gazetted to the important post of Principal of the Madras Medical College.

The death is announced of Dr. John Wilson. He studied at St. Bartholomew's and took his diploma in 1880, becoming an M.D. of St. Andrews in 1894. Dr. Wilson was district surgeon to the Midland Railway, and at one time medical attendant of the Harpenden Hall Asylum.

The death is announced, at Torquay, of Dr. Thomas Seccombe, M.D., F.R.C.S., late deputy inspector-general of hospitals and fleets at the age of 90. Dr. Seccombe took the degree of M.D. at Aberdeen in 1865, and became Member and Fellow of the R.C.S. in 1843 and 1864 respectively. He had retired from practice for some years, and made his home at Upper Norwood.

The death is announced at Billingford, Norfolk, of Dr. George Lichtenberg, aged eighty-three. Dr. Lichenberg took the degree of M.D. at Gottingen in 1851, becoming M.R.C.P. of London in 1860 and M.R.P.S. in 1863. Before retiring to the country Dr. Lichtenberg was consulting surgeon to the Training Hospital, Tottenham, and also a Fellow of the Hunterian Society. He held at one period the post of senior surgeon to the German Hospital.

